# A Multi-Agent Prediction Method for Data Sampling and Transmission Reduction in Internet of Things Sensor Networks

**DOI:** 10.3390/s23208478

**Published:** 2023-10-15

**Authors:** Bartłomiej Płaczek

**Affiliations:** Institute of Computer Science, University of Silesia, Będzińska 39, 41-200 Sosnowiec, Poland; bartlomiej.placzek@us.edu.pl

**Keywords:** sensor network, Internet of Things, transmission reduction, prediction model, multi-agent system

## Abstract

Sensor networks can provide valuable real-time data for various IoT applications. However, the amount of sensed and transmitted data should be kept at a low level due to the limitations imposed by network bandwidth, data storage, processing capabilities, and finite energy resources. In this paper, a new method is introduced that uses the predicted intervals of possible sensor readings to efficiently suppress unnecessary transmissions and decrease the amount of data samples collected by a sensor node. In the proposed method, the intervals of possible sensor readings are determined with a multi-agent system, where each agent independently explores a historical dataset and evaluates the similarity between past and current sensor readings to make predictions. Based on the predicted intervals, it is determined whether the real sensed data can be useful for a given IoT application and when the next data sample should be transmitted. The prediction algorithm is executed by the IoT gateway or in the cloud. The presented method is applicable to IoT sensor networks that utilize low-end devices with limited processing power, memory, and energy resources. During the experiments, the advantages of the introduced method were demonstrated by considering the criteria of prediction interval width, coverage probability, and transmission reduction. The experimental results confirm that the introduced method improves the accuracy of prediction intervals and achieves a higher rate of transmission reduction compared with state-of-the-art prediction methods.

## 1. Introduction

In the Internet of Things (IoT), various physical devices, actuators, sensor nodes, and other objects can communicate to automate processes in a variety of areas, including environmental monitoring, healthcare, transportation, farming, agriculture, payments, and smart grids [1,2]. Nowadays, many new IoT applications are impacting nearly all areas of everyday life. Wireless Sensor Networks (WSNs) are essential components of IoT systems, providing real-time information about the system and its environment [3,4]. With the widespread adoption of IoT applications, the amount of data being collected from sensor devices is increasing rapidly. As a result, there has been an enormous surge in the amount of data being generated and transmitted over the Internet [5]. However, there are significant limitations imposed by network bandwidth, data storage, processing capabilities, and the finite energy resources of battery-powered devices. Thus, methods are necessary to reduce the amount of sensed and transmitted data [6]. In this context, the research objective is to exclude irrelevant sensor readings without compromising the effectiveness of a given IoT application or service.

In this paper, the use of prediction schemes [7,8] is analyzed as a promising approach to addressing the problem of data sampling and transmission reduction in WSNs. Let us consider a sensor node in a WSN, which reports its data readings to a gateway. According to the analyzed approach, a gateway predicts future sensor readings to determine if the actual sensed data can be useful for a considered IoT application. For instance, the sensor readings are useful if they reveal that a monitored parameter has reached a level at which the IoT system triggers an action. Based on the prediction results, the time is determined for collecting and transmitting the next data sample. For the above-mentioned example, the prediction results are used to estimate the shortest time in which the monitored parameter can reach the level of interest. Then, the sensor node is put into sleep mode until the scheduled time, effectively reducing the communication load and saving energy. In this scenario, an appropriate prediction method is necessary to determine the range of possible future sensor readings.

The new prediction method introduced in this paper is based on a multi-agent system, where each agent provides its own forecast. The predictions are made using historical sensor readings that closely resemble the most recent data.

When making predictions, agents utilize distinct parts of the time series with historical data. Aggregated predictions from all agents determine the range of possible future values (prediction interval). To generate a forecast for multiple future time steps, the agents iterate through the time series of historical data and perform the prediction operation repeatedly. New agents are created when similar data sequences are found at different time series points. Agents can also be removed if they duplicate other agents or if they reach the end of the time series. This method was developed to achieve a high coverage probability and narrow width of the prediction intervals, which allows for the efficient reduction of data sampling and transmission in WSNs.

This paper presents a new prediction method that enables the IoT gateway to determine the optimal timing for collecting and transmitting data readings from sensor nodes. The proposed method is evaluated experimentally using real-world sensor data. The paper is organized as follows. The motivation and objectives of the research are presented in Section 2. Section 3 reviews the works related to prediction-based transmission reduction in WSNs. Section 4 presents the proposed multi-agent prediction method in detail. This method was verified through experiments and compared with state-of-the-art approaches, as described in Section 5. Finally, conclusions are included, and future research directions are suggested in Section 6.

## 2. Motivation and Objectives of Research

As was already mentioned in the previous section, this study focuses on the prediction-based method, which enables us to minimize data sensing and transmission from the sensor node to the gateway. According to this method, the gateway receives current sensor readings, makes a prediction, and determines the timing for the next transmission. Information about the next transmission time is sent to the sensor node. Then, the sensor node switches to sleep mode until the scheduled time, when the next data sample will be collected and transmitted.

The research objective is to find a prediction algorithm that allows the gateway to effectively schedule data transmissions from sensor nodes. The motivation for this research is the fact that the implementation scenario being considered imposes specific requirements on the prediction method.

- The prediction has to be made for multiple steps ahead.
- The intervals of possible values need to be predicted.
- The high coverage of prediction intervals is required.
- The width of prediction intervals should be minimized.

A multiple-step prediction is necessary because the time periods between subsequent transmissions have different lengths, and the sensor node can collect multiple data samples during these periods. A prediction of the most probable (expected) values is insufficient because the gateway requires information about all possible future sensor readings in order to promptly identify the situation when a monitored parameter reaches the level of interest. Thus, interval prediction is used in this scheme.

**Figure 1:**
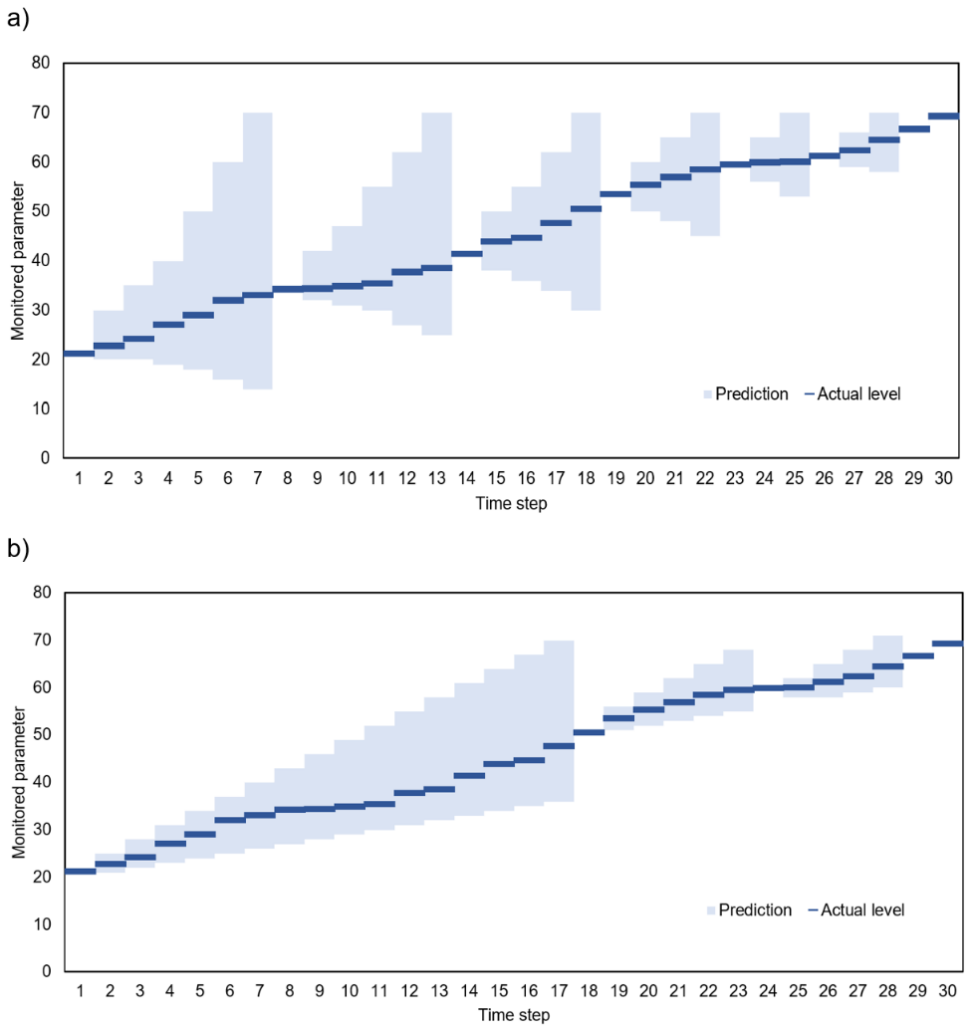
Examples of prediction-based data collection: (**a**) wider prediction intervals, (**b**) narrower prediction intervals.

Illustrative examples of the considered prediction-based data collection process are presented in Figure 1. For these examples, it is assumed that the IoT system has to perform an action when the monitored parameter exceeds a value of 70. In Figure 1a, the actual sensor reading is transmitted to the gateway at time step 1. Then, the gateway makes a prediction, which shows that the level of 70 may be exceeded at time step 8. The next transmission is scheduled for time step 8. The sensor node remains in sleep mode during steps 2–7, and sensor readings are not collected during this period. During this period, the sensor node is in a low-power state and does not engage in data collection or processing. At time step 8, the actual value of the monitored parameter is reported to the gateway, and further sensor readings are predicted. The procedure is repeated, resulting in subsequent transmissions performed at time steps 14, 19, 23, 26, 29, and 30. This example demonstrates that estimating prediction intervals for multiple steps ahead allows us to reduce the amount of sampled and transmitted data. Moreover, this approach ensures that the actual sensor readings are registered when the monitored parameter reaches the level of interest (70).

When comparing the examples in Figure 1a,b, it can be observed that the number of transmitted data is lower if the prediction intervals have a smaller width. Note that the width of prediction intervals in Figure 1a is larger than that in Figure 1b. The number of transmissions is eight in example (a) and five in example (b).

**Figure 2:**
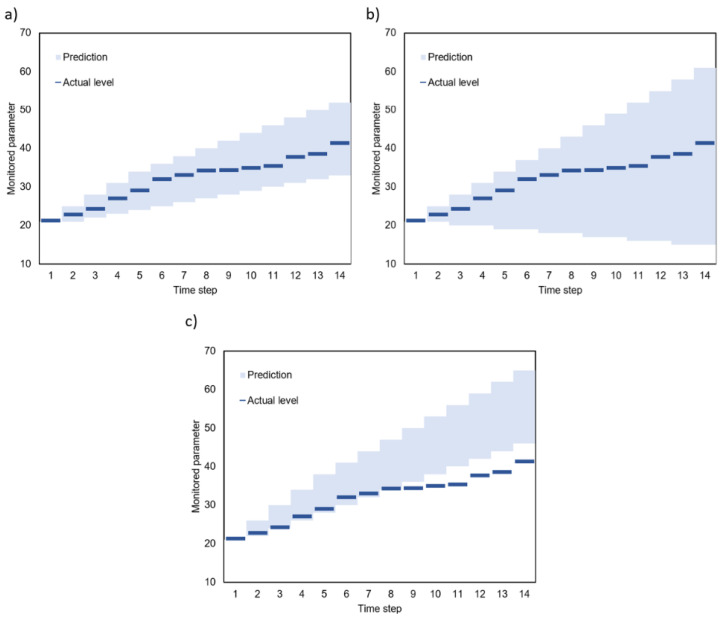
Examples of prediction results: (**a**) small width and high coverage, (**b**) large width and high coverage, (**c**) small width and low coverage.

The prediction results have high coverage if all (or almost all) actual values are inside prediction intervals. An example of prediction results with low coverage is shown in Figure 2c. The remaining examples in Figure 1 and Figure 2 present predictions with high (100%) coverage. In cases of low coverage, the actual value is outside the possible range determined by the gateway. This means that the operation of an IoT system can be incorrect (inadequate to the actual situation) if the real values of the monitored parameter are not taken into account. Thus, the low coverage is unacceptable when the prediction algorithm is used in the transmission reduction scheme being considered. Examples (a)–(c) in Figure 2 are ordered from best to worst case. The narrow intervals and high prediction coverage in example (a) ensure the correct operation of the IoT system and reduce data transmission effectively. The high coverage in example (b) ensures the correct operation of the IoT. However, the wide intervals result in a less efficient reduction in data transmission. Finally, in the case of narrow intervals and high prediction coverage (example (c)), the reduction in transmission can lead to incorrect operation of the IoT system.

To the best of the author’s knowledge, the applicability of the existing prediction algorithms has not been analyzed so far with regard to the aforementioned requirements.

## 3. Related Works

Various prediction methods have been proposed in the literature as effective tools for reducing data sensing and transmission rates in IoT-based WSNs [9,10]. The prediction techniques can be broadly categorized into two main approaches: single prediction and dual prediction.

In the case of the single prediction scheme, a prediction algorithm is implemented on the gateway [11,12]. The gateway can forecast sensor readings and determine when to collect real sensor data by taking into account the reliability of the current predictions. Alternatively, sensor nodes can make predictions to determine the expected changes in their environment. When the predicted changes are not significant, the sensor node may avoid unnecessary measurements and transmissions [13].

The single prediction scheme was used in WSNs to answer user queries without directly collecting the data from the sensor nodes [11]. According to that approach, each query determines the required data and the error tolerated by the user. The gateway indicates that the actual measurements fall within a range of values determined by the prediction results, provided that the prediction confidence is sufficiently high to meet the user-defined error. To this end, the gateway uses statistical models created with historical data [14] to evaluate the confidence level related to forecasts of current sensor readings and decide whether to collect more measurements from sensor nodes.

In [15], the single prediction method was implemented with a spatial-temporal correlation evaluation for sampling and transmission reduction in cluster-based WSNs, where the cluster head is responsible for collecting data from its member sensor nodes. The cluster head computes a correlation function in order to determine the degree of correlation among sensor nodes. Then, the sampling rate of the sensor nodes is adjusted according to the calculated correlation level. In order to ensure the integrity of the collected data, a prediction algorithm is deployed. This algorithm enables a base station to reconstruct the missing data points. During experiments, this method has enabled a reduction in energy consumption of up to 60%. Another approach to single prediction assumes that the operation of a WSN is scheduled with periods of data transmission followed by periods of data prediction [16]. Thus, the collection of actual measurements from sensor nodes is skipped during the data prediction period. The authors used a deep learning technique to create a prediction model that enables the cluster head to forecast sensor readings. It was shown that the application of the prediction model reduces up to 33% of the total energy needed by the WSN in an industrial IoT system [17].

The approach presented in [18] is based on implementing a prediction algorithm at the sensor node level. According to this method, the data readings collected by a sensor node during the first period are used to predict the sensor readings for the next period. The sensor readings from the first period are always transmitted to the gateway after being compressed. Then, during the next period, the similarity is calculated between the predicted data and the new sensor readings. If the similarity score is greater than or equal to a predefined threshold, the new data are not transmitted to the gateway.

In the dual prediction scheme, predictions are simultaneously made by the gateway and sensor nodes, all using the same prediction model. The sensor node compares the measured values with the prediction results and evaluates the prediction error. When the error falls below a predefined threshold, the sensor node does not transmit the actual data reading. Instead, the gateway utilizes its own predicted value in place of the actual sensor reading [19]. In this scheme, the predictions have to be made by the gateway and sensor nodes based on the same data sample [20].

To date, the dual prediction mechanism has been adapted for various application areas of IoT systems [9]. Faris et al. [21] applied this scheme to minimize data transmission between IoT sensor nodes and a medical server. In their solution, the sensor readings are reported to the server if predictions do not match the readings or if the data are considered critical based on predefined upper/lower limits or thresholds. Experimental results have confirmed that the method reduces energy consumption, extends the network’s lifetime, and ensures the required reliability, throughput, and end-to-end delay. In [22], it was shown that the efficiency of data transmission reduction in an IoT system significantly depends on the implemented prediction algorithm. The authors compared various prediction algorithms in terms of accuracy, delay, and percentage reduction in transmission. Furthermore, neural networks and long short-term memory networks have been suggested for dual prediction schemes with online model training.

A prediction method for content-based sensor node searches in IoT systems was introduced in [23]. It was demonstrated that the target sensor nodes can be quickly located by predicting the current output of the sensors. The dual prediction scheme was implemented in that system to update prediction models online. This approach allowed the prediction model to maintain its performance for long-term prediction in a dynamic IoT environment.

Another method, based on the dual prediction scheme, was introduced in [24] to address the issue of unreliable sensor data. This approach involves a data reduction phase and a data prediction phase. The objective of the data reduction phase is to decrease the number of transmissions and discard faulty sensor readings. In order to maintain data reliability, the discarded faulty data are then replaced with estimated values. During the data prediction phase, the non-transmitted sensor readings are predicted using the Kalman filter.

A deep learning technique was implemented with the dual prediction scheme in [25]. This scheme uses a long short-term memory network model to reduce the frequency of data transmission from connected IoT devices. The predictive model is updated based on a set of tracking parameters that monitor the model’s behavior during deployment, taking into account changes in data properties over time. The presented updating algorithm ensures that the models deployed at sensor nodes and the gateway are identical.

Even though many prediction techniques and models have been proposed to enable transmission reduction in WSNs, the methods for estimating prediction intervals have not yet been fully explored in this field. The existing transmission reduction methods are based on the assumption that the WSN has to deliver a point estimate of the sensor reading, such that the absolute difference between the point estimate and the real measurement is not greater than a given error bound. In contrast, this work assumes that the WSN can deliver an interval of possible (predicted) sensor reading values, and the target application decides if such information is sufficient. If not, then the actual sensor reading has to be transmitted. This approach allows the WSN to better fit the precision of the delivered information to the current application needs and opens new perspectives for improving the efficiency of transmission reduction. As discussed in Section 2, the efficient reduction of data transmission and sensing can be achieved when the prediction algorithm ensures high coverage and small width of the prediction intervals. Thus, this paper introduces a prediction method that meets these requirements and compares it with existing techniques for prediction interval estimation.

## 4. Proposed Method

This section presents details of the proposed multi-agent prediction method. The introduced method allows us to estimate intervals that include possible future values of a sensed parameter for multiple steps ahead. It should be noted that the agents in this method do not have a direct one-to-one relationship with specific sensor nodes. Instead, they collaborate to analyze historical data and identify patterns that enable them to predict future sensor readings for any sensor node within the network. This cooperative approach allows for more effective and accurate predictions by leveraging the historical data collected from the entire IoT sensor network.

Algorithm 1 shows the pseudo-code of the prediction procedure. Input data of this procedure include time series with historical sensor readings (*H*) and several recent measurements (*R*). The historical data *H* must be collected before executing the prediction algorithm. It should be noted that the time series *H* is significantly longer than *R* (n≫m). Parameter *T* determines the prediction horizon, i.e., the number of time steps in the future for which the intervals of possible sensed values ($P_{t}$) should be evaluated.

At the start of the multi-agent prediction process, agents are generated by evaluating the similarity between the sequence of actual measurements *R* and subsequences of the historical time series *H*. Details of agent creation are presented in Algorithm 2. When an agent is created, its birth time is set to $\tau_{i}$, indicating a data point in time series *H*, where the historical data are similar to the recent sensor readings *R*. The Euclidean distance is taken into account to assess the similarity between historical and current sensor readings. Thus, the birth times of agents are selected that correspond to the time points in *H* where the Euclidean distance is at its lowest. This is achieved by sorting the time points in increasing order according to the Euclidean distance and selecting the first several data points from the ordered set *B* (see lines 8–13 in Algorithm 2). The number of created agents is limited by $k_{\max}$ (maximum number of agents). However, $k_{\max}$ agents are created only if the relevant number of similar subsequences were found in *H* with the Euclidean distance to *R* not greater than α. Additionally, the parameter $k_{\min}$ was introduced to determine the minimum number of agents. A group of $k_{\min}$ agents is set up, even if the distances between the corresponding subsequences of historical data and *R* are greater than α. This approach ensures that the required number of agents is always available to make predictions. It should be noted that $k_{\max}$ is assumed to be greater than $k_{\min}$.

After agents are created, the operations in lines 4–15 of Algorithm 1 are repeated for all time steps of the prediction. The age of each agent is incremented. Then, the agent makes a prediction if its age is above 0. The prediction is determined as the historical sensor reading $h_{\tau^{*}}$ from *H*, where the index $\tau^{*}$ is equal to the sum of the agent’s age and birth time.**Algorithm 1** Multi-agent prediction 1:**Inputs:**  recent sensor readings *R* = {*r*_1_, … , *r_m_*}  historical sensor readings *H* = {*h*_1_, … , *h_n_*}  prediction horizon *T* 2:**Inputs:**  intervals of predicted sensor readings *P_t_* = [$p_{t}^{-}$, $p_{t}^{+}$], *t* = 1, … , *T* 3:Create agents 4:**for** each time step *t* = 1 …*T* **do** 5:    **for** each agent **do** 6:    Increment age 7:    **if** age > 0 **then** 8:       Make prediction 9:       Reproduce10:    **end if**11:    **end for**12:    Remove unnecessary agents13:    $p_{t}^{-}$: =min{agents’ predictions}14:    $p_{t}^{+}$: =min{agents’ predictions}15:**end for**

The existing agents with ages greater than 0 can also reproduce to create new agents. Details of the reproduction operation are shown in Algorithm 3. During this operation, subsequences of the historical data *H* are searched that are similar to the subsequence denoted by *V*. The considered subsequence (*V*) includes a prediction of the existing agent ($\tau^{*}$) and m−1 preceding data points from *H*. The similarity of the subsequences is evaluated by taking into account the Euclidean distance. When the distance is lower or equal to β, then a new agent is created (see line 9 in Algorithm 3).

The multi-agent prediction algorithm also includes the removal of unnecessary agents (Algorithm 1, line 12). Specifically, an agent is removed if it replicates the prediction of another agent (i.e., both agents have the same sum of birth time and age). Moreover, an agent is deleted when its prediction is beyond the scope of the time series with historical sensor readings (the sum of birth time and age is larger than *n*). The above criteria for agent removal are summarized in Equation (1). An agent *a* is removed if the following condition is satisfied:

(1) $${\mathrm{birth}\mathrm{time}}_{a}+{\mathrm{age}}_{a}>n\;\vee \;\exists b\in A:{\mathrm{birth}\mathrm{time}}_{a}+{\mathrm{age}}_{a}={\mathrm{birth}\mathrm{time}}_{b}+{\mathrm{age}}_{b},$$

where *a* and *b* are identifiers of the agents, *A* denotes the set of identifiers of all existing agents, and *n* is the length of the sequence that contains historical sensor readings.
**Algorithm 2** Create agents 1:**Inputs:**  recent sensor readings *R* = {*r*_1_, … , *r_m_*}  historical sensor readings *H* = {*h*_1_, … , *h_n_*}  distance threshold α  minimum number of agents *k*_min_  maximum number of agents *k*_max_ 2:**Onputs:**  set of agents *A* 3:*A* := empty set 4:*B* := {*m*, … , *n*}                  ▷ ordered set of time points 5:**for** each time point *τ_i_* ∈ *B* **do** 6:  *distance*(*τ_i_*) := Euclidean distance between {*h*_*τ_i_*−*m*+1_, … , *h*_*τ_i_*_} and *R* 7:**end for** 8:Sort time points *τ_i_* ∈ *B* in ascending order according to *distance*(*τ_i_*) 9:**for** *i* = 1 … *k*_max_ **do**10:  **if** *i* <= *k*_min_ or *distance*(*τ_i_*) <= *α* **then**11:   Add new agent to A with birth time = *τ_i_* and age = 012:  **end if**13:**end for**

Finally, the prediction interval for time step *t* is determined in lines 13 and 14 of Algorithm 1. The endpoints of this interval correspond to the minimum and maximum of the agents’ predictions.

**Figure 3:**
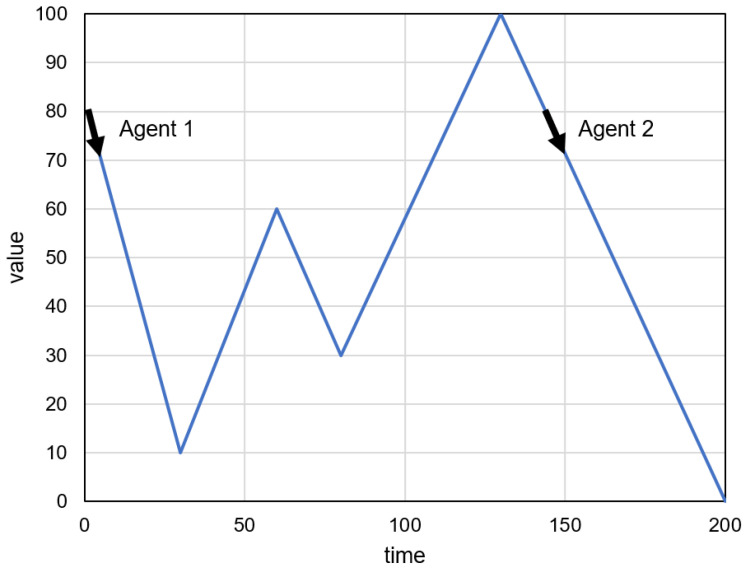
Example of the proposed algorithm operation: initially created agents.

**Figure 4:**
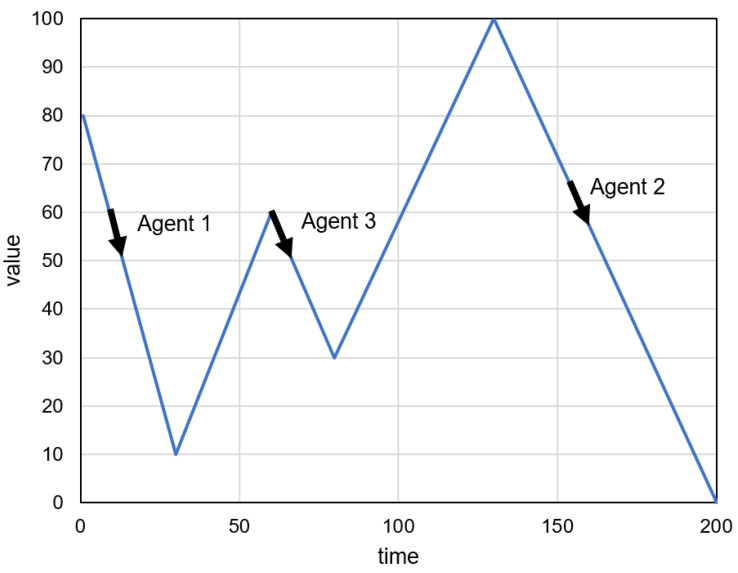
Example of the proposed algorithm operation: Agent 3 created as a result of reproduction.

**Figure 5:**
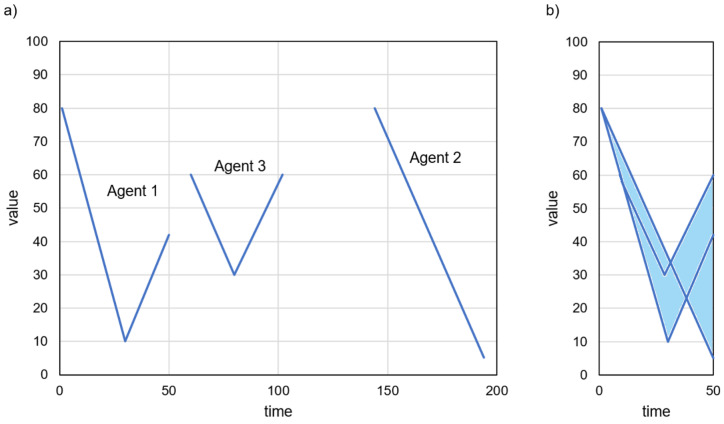
Example of the proposed algorithm operation: (**a**) individual predictions of agents, (**b**) resulting prediction intervals.

An illustrative example of the proposed algorithm operation is presented in Figure 3, Figure 4 and Figure 5. The prediction intervals are evaluated in this example for 50 time steps (T=50). It is assumed that the time series of recent sensor readings (*R*) establishes a decreasing trend starting at a value of 80. The historical sensor readings (*H*) are represented in Figure 3 and Figure 4 by the blue lines. Initially, the sequences similar to *R* are found at time points 1 and 144, and the agents are created with corresponding birth times (see the black arrows in Figure 3). The ages and predictions of the two agents are updated in subsequent iterations of the algorithm. Figure 4 depicts the state of the agents after nine iterations (t=9). The new agent (Agent 3) is created as a result of the reproduction of Agent 1. The results of the prediction obtained during 50 iterations for three agents are presented in Figure 5. Figure 5b shows the prediction intervals determined by aggregating the individual agents’ forecasts.
**Algorithm 3** Reproduce 1:**Inputs:**  historical sensor readings *H* = {*h*_1_, … , *h_n_*}  agent’s birth time and age  distance threshold *β* 2:**Onputs:**  Updated set of agents *A* 3:τ^*^ = birth time + age 4:*V* = {*h*_*τ_i_*−*m*+1_, … , *h*_*τ_i_*_} 5:**for** *τ* = *m*, … , *n* **do** 6:   **if** *τ* ≠ τ^*^ **then** 7:    *distance*(*τ_i_*) := Euclidean distance between {*h*_*τ_i_*−*m*+1_, … , *h*_*τ_i_*_} and *V* 8:    **if** *distance*(*τ*) <= *β* **then** 9:    Add new agent to A with birth time = *τ* and age = 010:    **end if**11:   **end if**12:**end for**

The analyzed example shows that the introduced agent reproduction enables us to determine prediction intervals that take into account situations where the predicted value for a specific time step may result in multiple different values at subsequent time steps. An example of such a situation can be observed in Figure 5, where the line branching is observed for time = 9 and value = 60.

## 5. Experiments and Discussion

The experiments were conducted to evaluate the effectiveness of the proposed method and demonstrate the benefits of using prediction intervals for reducing data transmission in WSNs. During the experiments, the criteria of prediction interval width, coverage probability, and transmission reduction were considered to verify if the introduced method satisfies the requirements discussed in Section 2 and contributes to reducing the amount of transmitted data. Received Signal Strength Indicator (RSSI) data from a mobile device were used to evaluate the multi-agent prediction scheme experimentally. The experiments also involved comparison with state-of-the-art methods that can be used to predict the intervals of future sensor readings. Moreover, an example of a signal strength monitoring application was considered to assess the effect on transmission reduction.

### 5.1. Dataset and Evaluation Criteria

**Figure 6:**
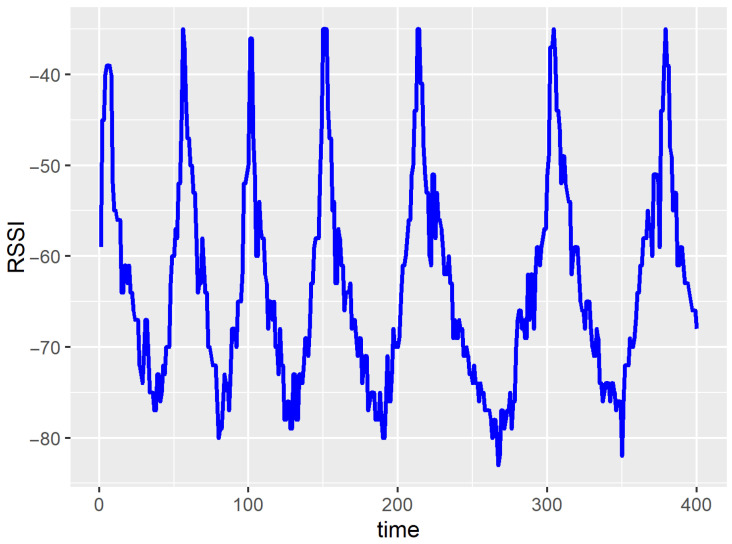
Example of RSSI data.

The RSSI time series dataset were collected using a mobile device (smartphone) connected to a WiFi access point and a network signal monitoring application. The smartphone was kept by a person walking in an indoor environment. The sampling period for the RSSI measurements was 2 s. While moving at varying speeds, the distance between the mobile device and the access point was alternately increased and decreased, resulting in fluctuations in the RSSI value. An example of the RSSI time series is presented in Figure 6. It should be noted that the periodicity is not constant in these data, as the person’s speed and route changed during the measurements. The dataset used in this study contains 23,180 RSSI values.

The use of a smartphone and WiFi access point during data collection was for experimental convenience and does not define the scope or limitations of the proposed method. It is important to emphasize that these devices do not establish the intended platform for implementing our proposed method. This method can be readily adapted to sensor networks with low-end IoT devices, and the sensing tasks can be tailored to suit the specific needs of the application. The introduced method can be effectively applied to various sensor node configurations and sensing tasks within the realm of IoT sensor networks, including different communication technologies.

The effectiveness of the examined prediction methods was evaluated by considering the criteria of prediction interval width and coverage. As discussed in Section 2, these criteria are crucial in situations where prediction methods are used for sampling and transmission reduction. Detailed definitions of the evaluation measures are based on those used in [26]. The prediction interval coverage probability (PICP) and the normalized mean prediction interval width (NMPIW) were calculated using the following formulas:

(2) $$\mathrm{PICP}=\frac{1}{N_{t}}\sum_{i=1}^{N_{t}}B_{i},$$

(3) $$\mathrm{NMPIW}=\frac{1}{RN_{t}}\sum_{i=1}^{N_{t}}\left(p_{i}^{+}-p_{i}^{-}\right),$$

where $N_{t}$ is the number of tested data points; $B_{i}$ denotes a Boolean variable, such that $B_{i}=1$ when the *i*-th data point belongs to the predicted interval $\left[p_{i}^{+},p_{i}^{-}\right]$, otherwise $B_{i}=0$; and *R* denotes the range of observed RSSI values.

For a considered example of a WSN application, the effectiveness of the compared methods was also assessed by analyzing the data transmission reduction rate (DTRR), which is defined as

(4) $$\mathrm{DTRR}=\frac{DT-DTR}{DT},$$

where DT is the number of data transmissions without reduction (achieved when the transmission is performed at each step of the WSN operation), and DTR is the number of transmissions executed when the prediction-based reduction is implemented.

### 5.2. Compared Methods

Representative state-of-the-art prediction methods were selected for the experimental comparison based on a literature review [27,28]. The methods that allow us to evaluate prediction intervals for multiple steps ahead were considered. The selected techniques are based on various formal frameworks, including Monte Carlo simulation, bootstrapping, machine learning, and statistical modeling.

The naïve algorithm [29] takes one past value and uses it as the predicted value. The naïve method evaluates prediction intervals for a random walk model, which is equivalent to the Autoregressive Integrated Moving Average ARIMA(0,1,0) model. The model assumes that the forecast distributions are normal. Thus, the conditional variance is calculated for the normal distribution in order to obtain prediction intervals.

The second method utilizes a feed-forward neural network with a single hidden layer and lagged inputs [30]. The neural network model was fitted to the RSSI time series with lagged time series values as inputs. The optimal number of lags was selected according to the Akaike Information Criterion (AIC). The number of neurons in the hidden layer was equal to half of the number of lags plus one. The neural network was trained for one-step prediction, and the multi-step forecasts were computed recursively. This approach establishes an autoregressive model. Thus, it is not possible to derive prediction intervals analytically. Instead, the prediction intervals were calculated through simulations. Future sample paths of this model were generated iteratively by randomly generating an error value from a normal distribution. The number of simulations was set to 1000. Prediction intervals were determined in this method by using the fitted neural network and repeatedly simulating sample paths.

Next, a method was compared that uses the bootstrap technique [31] to evaluate prediction intervals. The general concept of bootstrapping is to resample the random component of a time series in order to create a new series that retains the same underlying patterns (trends) but has a different remainder. Then, multiple predictions are made using the original and the bootstrapped time series. The results of these forecasts are then aggregated in the prediction interval. The implemented bootstrap approach [32] involves decomposing the time series into trend and remainder via LOESS (locally estimated scatterplot smoothing) transformation. The remainder is bootstrapped to generate new vectors of remainders that follow the empirical distribution of the original remainder vector. The new vectors of remainders are added to the trend component to create a new bootstrapped series. The original and the bootstrapped time series are then extrapolated using the k-nearest neighbor forecast algorithm [33]. In this way, many predictions are produced, allowing us to evaluate the prediction intervals.

Finally, the Markov chain Monte Carlo (MCMC) method was implemented based on the approach presented in [34]. A Markov chain is a mathematical model that describes transitions from one state to another between a finite number of possible states [35]. To apply the Markov chain to synthetic RSSI time series generation, different states correspond to the integer RSSI values between −90 and −30 dBm. The probability for transitions among states is determined in the transition matrix. The second-order transition matrix was used, describing the next value’s probability based on the current and preceding values [36]. Synthetic time series were generated according to the information provided in the transmission matrix for a given initial state. A set of 1000 synthetic time series was used to evaluate the prediction intervals.

All examined methods were implemented in the R programming language [37]. The parameters of the compared method and those of the proposed algorithm were tuned during the preliminary experiments. The parameter settings were selected for each method to achieve high coverage of the prediction intervals and minimize the average interval width. The operations of both the IoT gateway and the sensor nodes were tested within the R Studio environment, which facilitates the training and testing of the prediction models. The algorithms were not implemented in a physical IoT gateway.

### 5.3. Experimental Results

The prediction experiments were conducted 100 times for all methods. During each experiment run, a subsequence of 50 data points were selected from the RSSI time series. This subsequence was used as the test data to calculate the PICP and NMPIW measures. The remaining part of the dataset was available as input for the prediction algorithms (training data). The prediction horizon was set to 50 time steps. The main objective of the experimental results analysis is to verify if the proposed approach can improve the quality of prediction intervals and reduce the amount of transmitted data in comparison with state-of-the-art methods.

**Figure 7:**
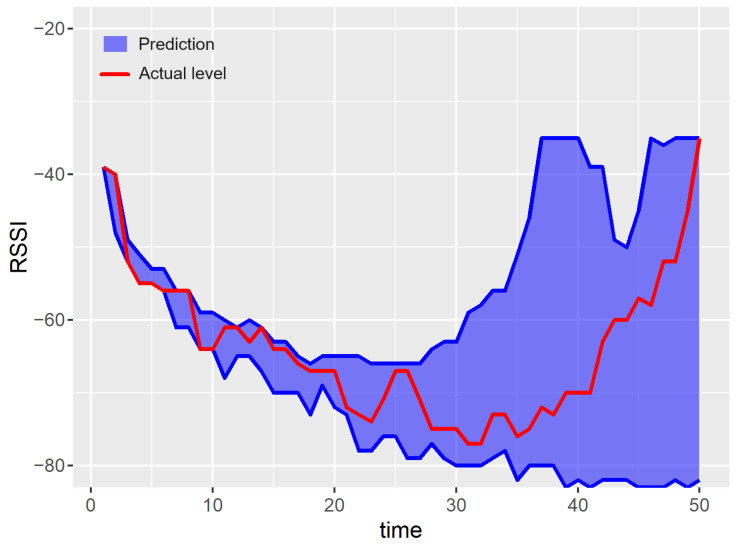
Results of the prediction interval estimation with the proposed multi-agent method.

**Figure 8:**
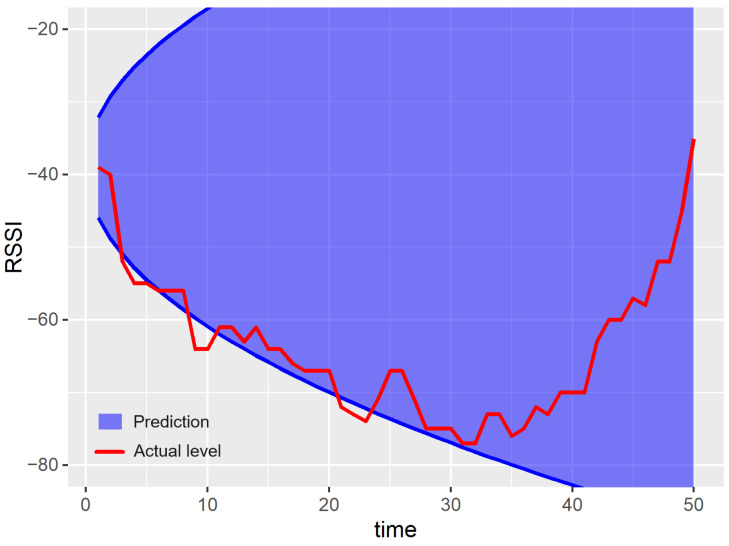
Results of the prediction interval estimation for the naïve method.

**Figure 9:**
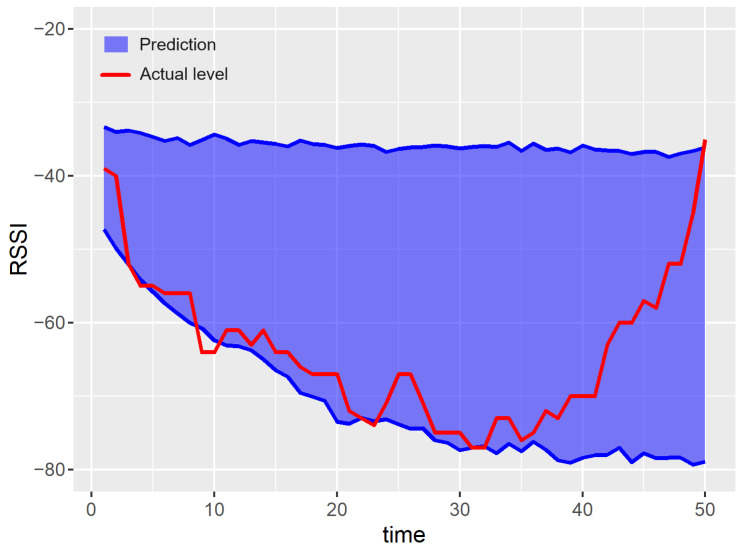
Results of the prediction interval estimation with the neural network model.

**Figure 10:**
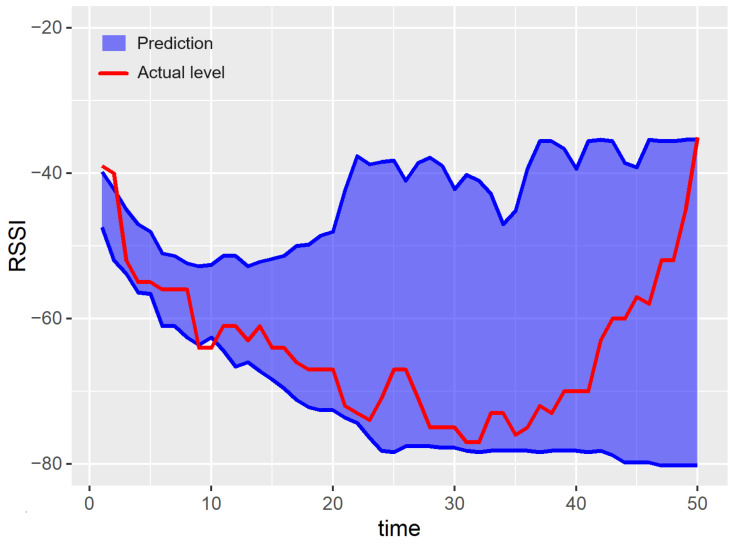
Results of the prediction interval estimation with the k-nn algorithm and bootstrapping.

**Figure 11:**
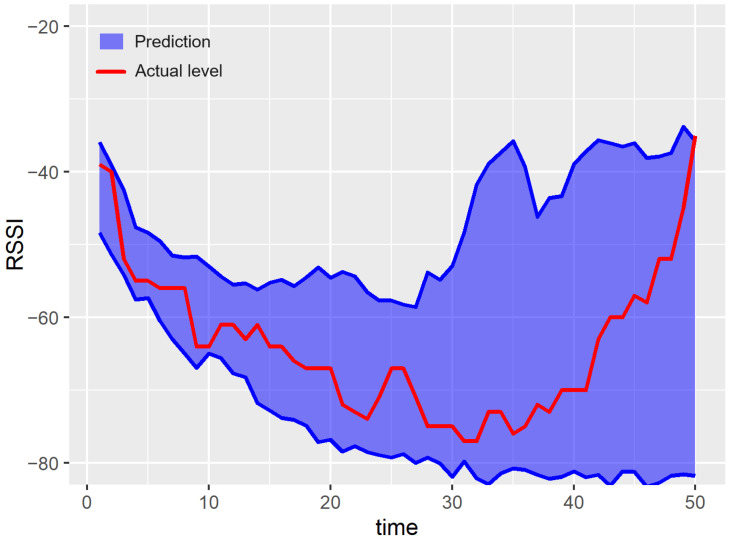
Results of the prediction interval estimation with the Markov chain Monte Carlo method.

Examples of prediction results for a single run of the experiment are shown in Figure 7, Figure 8, Figure 9, Figure 10 and Figure 11. The blue areas in these plots correspond to the evaluated prediction intervals, and the red lines show the actual RSSI values. Based on the presented results, one can visually assess the coverage and width of the obtained prediction intervals. The coverage is maximal if all the real measured values (represented by red lines) are within the blue region. Some evident cases of non-perfect coverage can be observed for the naïve algorithm, neural network, and bootstrapping method (see the predictions for the 10th time point).

The average interval width corresponds to the area of the blue region in Figure 7, Figure 8, Figure 9, Figure 10 and Figure 11 (the larger the area, the higher the average width of the prediction interval). The proposed algorithm and the MCMC method achieve significantly narrower prediction intervals, particularly for time steps 1–35. These models correctly anticipate the decline in the RSSI at the beginning of the prediction period. According to the analyzed dataset, the RSSI does not remain at the highest level (close to −40 dBm) for a long time. Thus, in the case of the naïve and neural models, the upper bounds of prediction intervals are evaluated unrealistically.

For both the proposed approach and the MCMC method, the width of the prediction interval is increasing slowly for time steps 1–30. Then, a rapid growth in the prediction interval width is observed. The reason behind these results is that the historical dataset includes examples where the RSSI values around −65 dBm are followed by an increasing trend. These cases are taken into account by the prediction algorithms when evaluating the upper bound of possible future RSSI values for the time steps 30–50.

The naïve algorithm did not correctly evaluate the upper bound of the prediction intervals. The results suggest that the RSSI could take values above −30 dBm, which is impossible. Thus, for further analysis, the prediction intervals returned by the algorithm were automatically reduced to the possible range of RSSI values ([−90, −30]). It means that the results outside the above-mentioned range were ignored when calculating the NMPIW metrics.

The linear forecasting methods, based on simple statistics such as the naïve algorithm, assume that the width of the prediction interval has to increase with the prediction horizon. This assumption leads to a higher NMPIW. In contrast, the non-linear methods (e.g., MCMC, bootstrapping, and the proposed algorithm) do not involve such limitations. Thus, the prediction intervals for these methods can be better fitted to the historical data. This effect can be observed in Figure 7 for time instances between 40 and 45.

In the case of the proposed approach, multiple agents extensively explore the historical (training) dataset to find possible future RSSI trajectories (sequences). This method builds up the prediction intervals by combining the relevant signal trajectories from historical data. Thus, unrealistic results are not generated. The results presented in this section were obtained with the following parameter settings of the proposed algorithm: prediction horizon T=50, distance threshold α=5, minimum number of agents $k_{\min}=3$, maximum number of agents $k_{\max}=10$, distance threshold β=4. The above values were selected based on preliminary experiments.

The quality of the prediction results delivered by the bootstrapping and MCMC methods strongly depends on the underlying procedures that generate synthetic time series. Thus, a careful calibration of these algorithms was necessary to accurately reproduce the trends observed in the historical data. In the case of MCMC, the first-order Markov chain model failed to provide satisfactory results. Thus, the higher-order model was used, which takes into account several previous values when randomly generating the next data point. During tests of the bootstrap approach, simple random replacement was found to be unsuitable for generating realistic vectors of reminders. Therefore, the block bootstrapping method [38] was implemented.

**Figure 12:**
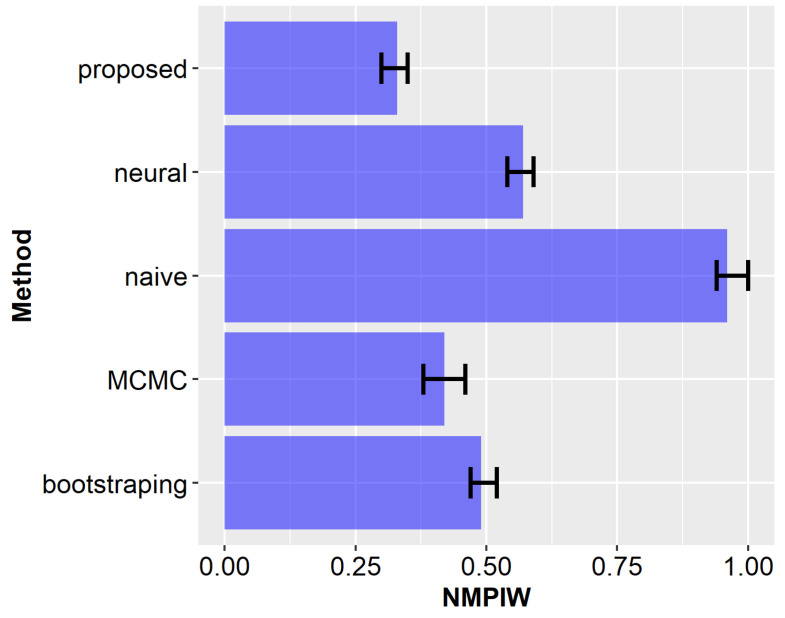
Comparison of normalized mean prediction interval width for the analyzed methods.

**Figure 13:**
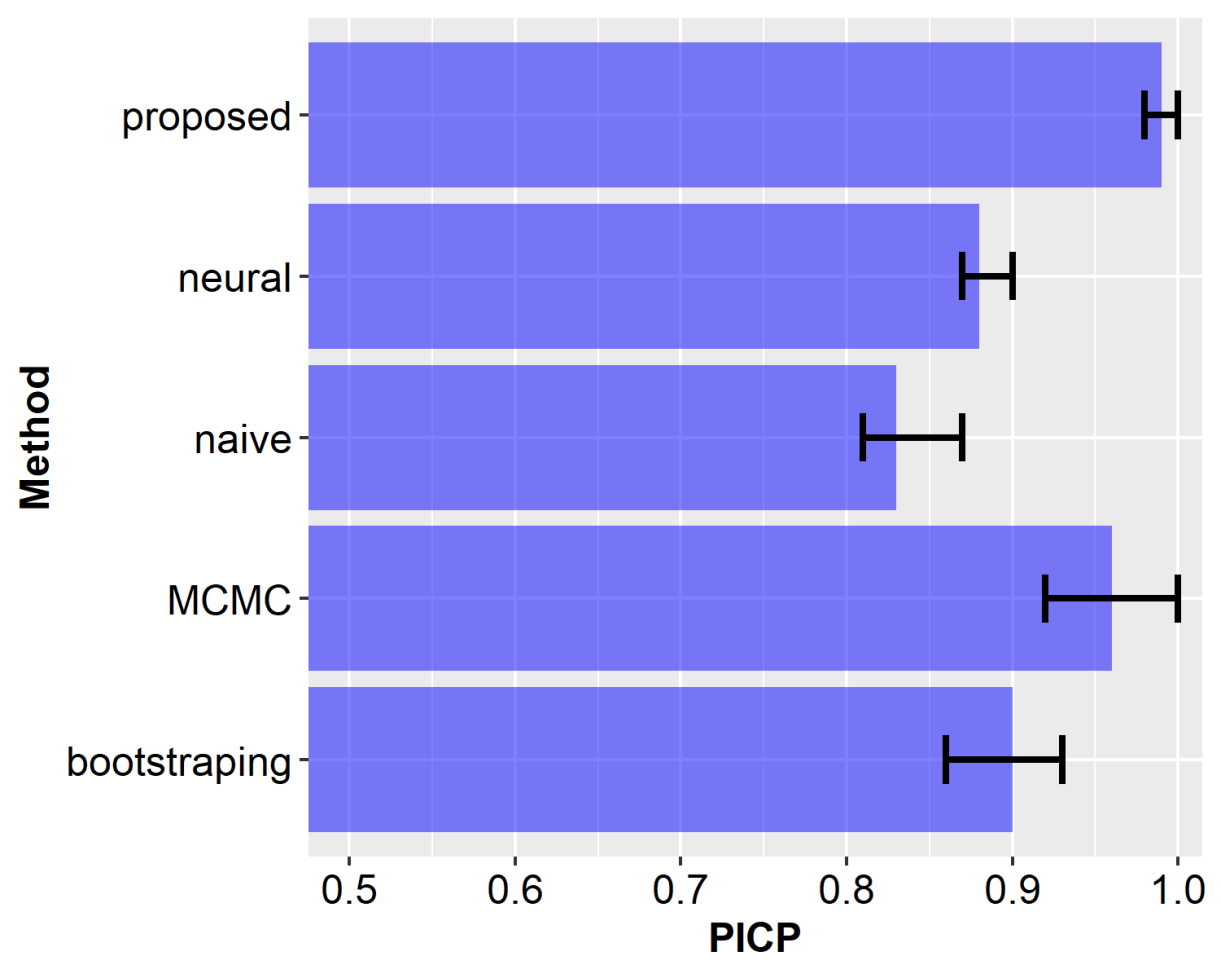
Comparison of prediction interval coverage probability for the analyzed methods.

**Figure 14:**
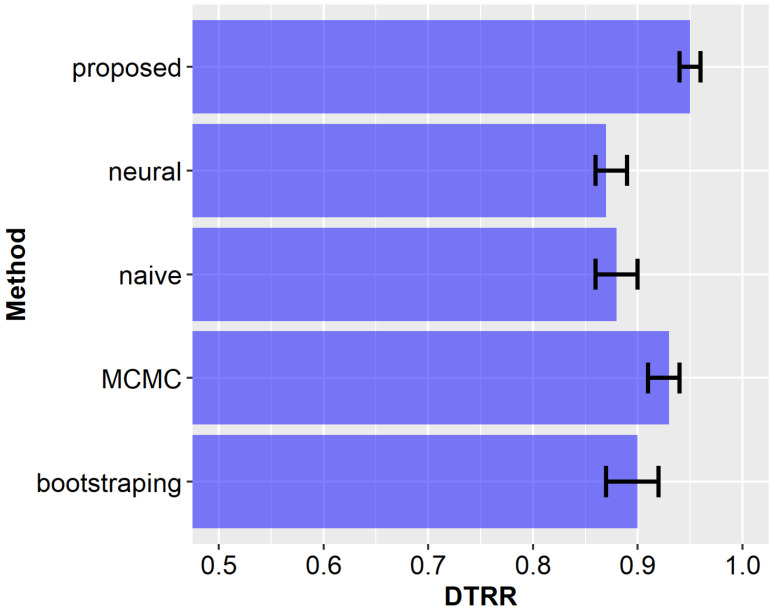
Comparison of transmission reduction for the analyzed methods.

Aggregated results (NMPIW and PICP) obtained after all runs of the experiments are compared for the examined methods in Figure 12 and Figure 13. The bars in Figure 12, Figure 13 and Figure 14 correspond to mean values, with error bars indicating the 5th and 95th percentile values obtained from 100 experimental runs. It can be observed from these results that the proposed method achieves a narrow width of the prediction interval and ensures a high coverage. For the remaining compared methods, the results are worse, i.e., they have lower coverage and larger prediction intervals. Among the state-of-the-art methods, the most promising outcomes were achieved by using the MCMC approach. However, the average NMPIW value for this approach increased by 0.09, while the average PICP decreased by 0.03. The worst results were achieved by the naïve algorithm, which is considered the baseline approach for comparison.

As is demonstrated in Section 2, the low interval width and high coverage of the prediction allow us to achieve a superior level of data sensing and transmission reduction. In this section, an example of an IoT system is considered to quantitatively asses the improvements in data reduction. It was assumed that a sensor node sends the RSSI readings to a gateway at selected time steps. The objective is to determine whether the RSSI value is below or above the threshold of −50 dBm. Thus, the gateway predicts the intervals of future RSSI values for multiple steps ahead and checks if the obtained intervals include the threshold value. On this basis, the next data sensing and transmission time is determined that corresponds to the minimum predicted time in which the RSSI can reach the threshold level. Up to this time, the data transmission operations are skipped. Then, several recent measurements are reported to the gateway to enable performing the next prediction, and the process is repeated.

The above scenario was analyzed during experiments, and the number of transmissions was evaluated for each of the compared prediction methods. The results of this analysis are presented in Figure 14. In the case of the proposed approach, 95% of the transmissions were eliminated (average DTRR = 0.95). This result improves the transmission reduction rates achieved by state-of-the-art prediction methods. The average DTRR of the MCMC method was 0.93. It should also be noted that state-of-the-art methods have a lower coverage probability. Thus, the information collected using those methods is less accurate. Another interesting observation is that the neural network model has a worse DTRR result than the naïve algorithm. The main reason is that in the case of naïve approach, narrower prediction intervals are obtained for short prediction horizons.

## 6. Conclusions

This paper analyzes the possibility of reducing the amount of sampled and transmitted data in IoT WSNs based on prediction interval evaluation. It was shown that prediction methods must meet specific requirements in order to effectively eliminate unnecessary data transmission and sampling. A new method was introduced to determine the intervals of possible sensor readings for many steps ahead with high coverage and low width. The proposed method is based on a multi-agent system, where each agent independently explores a historical dataset and evaluates the similarity between past and current sensor readings in order to make predictions. If the agent discovers multiple potential paths for future sensor readings, it generates new agents that take into account all of the possibilities. It has been demonstrated that the predicted intervals of possible sensor readings can be effectively used to suppress unnecessary transmissions and reduce the amount of data samples collected by the sensor node.

The experimental comparison with state-of-the-art prediction methods shows that the proposed approach determines narrower prediction intervals with a coverage probability close to one. This means that the prediction results describe the possible future sensor readings more accurately. The experiments also confirmed that the introduced prediction method allows us to achieve a higher rate of transmission reduction compared with the other methods. The potential directions for future research include extending the method to make multivariate predictions and designing heterogeneous agents that can combine different prediction algorithms into one system. Another important direction for future research involves validation of the presented approach with the use of real-world sensor network environments and IoT hardware.

## Data Availability

The dataset analyzed in this study is publicly available after contact with the correspondence author.
